# The social supergene dates back to the speciation time of two *Solenopsis* fire ant species

**DOI:** 10.1038/s41598-020-67999-z

**Published:** 2020-07-14

**Authors:** Pnina Cohen, Eyal Privman

**Affiliations:** Department of Evolutionary and Environmental Biology, Institute of Evolution, University of Haifa, Haifa, Israel

**Keywords:** Evolution, Genetics, Molecular biology

## Abstract

Colony social organization of multiple *Solenopsis* fire ant species is determined by a supergene with two haplotypes *SB* and *Sb*, which are similar to X/Y sex chromosomes. The ancestral monogyne (single-queen) social form has been associated with homozygous *SB*/*SB* queens, while queens in colonies with the derived polygyne (multi-queen) social structure are heterozygous *SB*/*Sb*. By comparing 14 *Solenopsis invicta* genomes and the outgroup *S. fugax*, we dated the formation of the supergene to 1.1 (0.7–1.6) million years ago, much older than previous estimates, and close to the estimated time of speciation of the two socially polymorphic species *S. invicta* and *S. richteri*. We also used 12 *S. invicta* and *S. richteri* genomes to compare the evolutionary distances between these species and the distances between the social haplotypes, and found them to be similar. A phylogenetic analysis suggested that the monophyletic *Sb* clade is more closely related to *S. richteri SB* haplotypes than to *S. invicta SB* haplotypes. We conclude that the formation of the supergene occurred concomitantly with the process of speciation of the *Solenopsis* socially-polymorphic clade, and hypothesize that the *Sb* variant first arouse in one incipiently-speciating population and then introgressed into the other populations or species.

## Introduction

Many ant species are socially polymorphic, displaying multiple alternative social structures. A key distinction between the social morphs is the number of egg-laying queens in a colony. In monogyne colonies, only one queen reproduces, making the colony a single family unit. Polygyne colonies, on the other hand, consist of multiple reproducing queens, which are not necessarily related^1,2^. Social polymorphism has been observed in at least six closely related South-American fire ant species^3–5^, informally known as the *Solenopsis* socially-polymorphic clade. The clade includes the red imported fire ant and the black imported fire ant, *Solenopsis invicta* and *Solenopsis richteri*, that were introduced to North America early in the twentieth century^6–8^.

In *S. invicta*, the monogyne/polygyne social structure is a Mendelian trait. It is linked to the gene *gp-9*, which codes for an odorant binding protein^3,9,10^. All and only monogyne queens are homozygotes *gp-9*^*BB*^, whereas reproductive polygyne queens are almost exclusively heterozygotes *gp-9*^*Bb*^ and very rarely *gp-9*^*bb*^^11–14^. *Gp-9* was thereby implicated as the molecular regulator of the polygyne social structure^5,15^.

More recently, it was suggested that *gp-9* was only one gene in a complex of genes that control social polymorphism^14,16^. Using a genetic map, Wang et al.^17^ showed that a non-recombining region of approximately 13.8 megabases, containing *gp-9* and over 500 additional genes, is associated with the social dimorphism of *S. invicta*. The suppression of recombination, probably caused by multiple chromosomal inversions, linked alleles of these genes to the *SB* and *Sb* haplotypes of a supergene, appropriately named the ‘social chromosome’. Over time, the two haplotypes diverged, with the *Sb* variant evolving separately from the *SB* variant, in a similar fashion to the differentiation taking place between X/Y sex chromosome pairs. While *SB* haplotypes can still recombine in *SB*/*SB* queens (as do the X chromosomes), *Sb* haplotypes no longer recombine. This resulted in the accumulation of deleterious mutation and repetitive elements in *Sb*, as in Y chromosomes^17–19^. In comprehensive molecular analyses, each of the six known socially polymorphic *Solenopsis* species was found to harbor two haplotypes of the supergene^19,20^. Moreover, the *Sb* haplotype was found to form a monophyletic clade, suggesting it is a derived haplotype that has evolved in a common ancestor of these species^20^.

While it seems reasonable to assume that the divergence of the *SB* and *Sb* variants predates the divergence of the species in the *Solenopsis* socially-polymorphic clade, it is also possible that historic admixture carried the polygyne variant across species boundaries. Such an event may have been driven by adaptive introgression as suggested for other loci of high fitness effect^21–24^. In a previous study, we used a coalescent-based analysis to date the separation of two social polymorphic species, *S. invicta* and *S. richteri*, to 1.1 (0.6–2.1) million years ago, assuming generation time of 6 years^25^. Additionally, we found no evidence of historic admixture between them, despite the large scale genomic data that was analyzed. This observation is a stark contrast with the prevalent hybridization that is taking place in their introduced range of South-Eastern USA^26,27^. At odds with this evidence, the divergence of the two social variants was dated to 390,000 (95% confidence level 350,000–420,000) years ago, much later than *S. invicta* and *S. richteri* ‘s estimated speciation time. This dating was calibrated by the divergence time of two species of leafcutter ants^17^, which were the most closely related species to the *Solenopsis* clade for which genomic sequences were available at the time.

As of late, more and more ant genomes are being sequenced, including the genome of the thief ant *Solenopsis fugax*^28^. The divergence of *S. fugax* and *S. invicta* was dated to 25 (18–32) million years ago (MYA) using 27 fossil calibration points, in a phylogenetic study that included 251 species in the Myrmicinae sub-family^29^. For comparison, in the same study the Attini lineage, that includes the two leafcutter ants, was found to have diverged from the *Solenopsis* lineage approximately 80 MYA. Additionally, reduced genomic sequencing of a large number of *S. richteri* samples is now available^25^, allowing the studying of the social chromosome outside of *S. invicta*.

Here, we take advantage of these new genomic data in order to study the appearance of the polygyne social structure, the evolutionary event that defines the *Solenopsis* socially-polymorphic clade. We measured the evolutionary distances between seven *SB* and seven *Sb* haplotypes in *S. invicta* individuals of both social variants, and, by comparing them to the homologous haplotype in *S. fugax,* dated the divergence of *SB* and *Sb* haplotypes to the time in which *S. invicta* and *S. richteri* speciated. A phylogenetic analysis placed *S. richteri SB* haplotypes in the same subtree as the *Sb* haplotypes of both species, suggesting the *Sb* variant first evolved in *S. richteri* and then introgressed into *S. invicta*. These results demonstrate that the dynamic evolutionary path of *Solenopsis* social chromosome is intertwined with a complicated history of diverging *Solenopsis* populations ever since it first appeared.

## Methods

### Aligning social chromosome haplotypes of fully sequenced S. invicta

Using SOAPdenovo2^30^, we de novo assembled 14 whole-genome sequenced haploid *S. invicta* males: seven *SB* variants and seven *Sb* variants, which were seven pairs of sons from seven unrelated *SB*/*Sb* queens collected in Athens, Georgia^17^. The sequencing data for the 14 samples were generated by Wang et al. using short-insert, paired-end, 100 bp reads Illumina sequencing. Our 14 assemblies had an average scaffold N50 size of 9,481 bp (range of 7,010–18,276 bp). We aligned these and three more genome assemblies: the reference genomes of *SB* and *Sb* variants of *S. invicta* (^28^ version Si_gnH; NCBI accession AEAEQ02000000) and the reference genome of *S. fugax* (genome assembly version Sf_gnA; NCBI accession QKQZ00000000) as an outgroup.

Using the reference *SB* genome as the subject, we ran blastn with default parameters (word_size = 11, gapopen = 5, gapextend = 2, reward = 2, penalty = -3) and aligned to it the scaffolds of the other 16 genome assemblies as queries. We then looked for genomic scaffolds in the 16 genomes that were aligned to the non-recombining region of the social chromosome located in chromosome 16 in the reference *SB* genome (scaffold00008; scaffold00028; scaffold00042; scaffold00045; scaffold00067; scaffold00119; 11,118,233 base-pairs in total). We only accepted alignments with E value ≤ 1 × 10^–4^ for the top hit. In order to avoid false inference of substitutions from alignments between different copies of repetitive sequences, we rejected alignments for which the second best hit, if such existed, either had E value <  = 1 × 10^–4^, or had half the number of base matches as the top hit or more, or had half the percentage of mismatches as the first hit or less. This resulted in a list of scaffolds of the 16 genomes which had homology and unique mapping to the social chromosome in the *SB* reference genome.

Next, we aligned the chosen scaffolds between these genomes, including the scaffolds of the non-recombining region in the reference *SB* genome, using Mugsy^31^, a multiple sequence aligner for whole-genome sequences. This resulted in 4,673 separate alignments blocks, each including sequences from all 17 genomes. We filtered out alignments that were less than 60 bp long and improved the remaining alignments further by running GUIDANCE2^32^ with the alignment algorithm PRANK^33^ and 50 bootstrap repeats. We masked bases that had GUIDANCE2 confidence scores of less than 60%. Eventually, we were left with 4,035 17-way alignments of a total length of 10,326,783 bp (including gaps and ambiguous bases), covering 7,093,489 bases in the non-recombining region, as it was identified in the reference *SB* genome, which is over 50% of its estimated total length.

We calculated pairwise distances between the 14 *S. invicta* genomes (not including the two *S. invicta* reference genomes) and the *S. fugax* genome by counting the number of substitutions in the alignments and correcting for multiple substitutions in the same site using the Jukes and Cantor (1969) method. We then calculated the distances between each pair of genomes. As a point of reference, we repeated the process detailed above using scaffolds that were aligned to chromosome 16 outside of the non-recombining region of the reference genome (i.e. not linked to *gp-9*).

### Aligning social chromosome haplotypes of RAD sequenced *S. invicta* and *S. richteri*

This analysis was based on Restriction site Associated DNA sequencing (RAD-seq) data of *S. invicta* and *S. richteri* populations samples from Argentina, which we described in Cohen and Privman^25^. We used sequences of four homozygote *SB*/*SB* and four homozygote *Sb*/*Sb richteri* workers from the population of Buenos Aires, Argentina. We also arbitrarily chose four homozygote *SB*/*SB invicta* workers from the population of El Recreo, Argentina. We mapped the RAD-seq samples to the reference genome of *S. invicta SB* using Bowtie2^34^. The results were filtered so that each alignment had no more than 4 mismatches for its top hit. To make sure we were using unique alignments only, we removed alignments that had for their second best hit double the number of mismatches as the top hit, or less. We then looked for loci in the non-recombining region in the *invicta SB* reference genome that had all 12 RAD-seq samples and the *invicta Sb* reference genome aligned to them. At each locus, we arbitrarily picked one read from each sample. We calculated distances between the 12 individuals by counting the number of substitutions between them, and applied Jukes and Cantor correction as above. As before, we repeated the analysis with loci outside the non-recombining region.

### Constructing phylogenetic trees for both datasets

Finally, using RAxML^35^ with the GTRCAT model, we built two maximum likelihood trees for the sequences of the non-recombining region in the samples in both datasets (whole genome and RAD-seq). In both cases *S. fugax* reference genome was used as an outgroup and was aligned to the *S. invicta* reference genome using Mugsy. For the RAD-seq dataset, we also mapped one of the RAD-seq *S. invicta* samples to the reference genome of invicta *Sb*, which allowed us to add this sequence to the multiple sequence alignment and to the RAxML phylogeny. The confidence in the trees were assessed using 100 bootstrap replicas.

### Tajima’s D anlalysis of haplotype structure

Tajima’s D^36^ is used to test a null hypothesis of neutral selection in a population of stable size based on the frequency of rare alleles in the population. Significantly negative (an excess of rare alleles) or positive (deficiency in rare alleles) values mean that the null hypothesis can be rejected. Negative and positive values can be caused by selective pressures or by changes in population size, such as sudden contraction and/or expansion during or after a population bottleneck.

Raw reads of the 14 fully sequenced *S. invicta* males were analyzed to create a catalogue of single nucleotide polymorphism (SNP) that was used in the analysis. Duplicated reads were removed (i.e., multiple read pairs that were identical in their sequence) and low quality reads were trimmed or removed using Trimmomatic^37^, leaving sequences of at least 75 bp. The catalogue was then created using HaplotypeCaller and GenotypeGVCFs of Genome Analysis Toolkit (GATK pipeline)^38^. We filtered out loci that had less than 6 reads in more than one individual. Additionally, we used a SNPs catalogue that was previously created from the RAD-sequencing of the 4 *SB/SB* and 4 *Sb/Sb* samples of *S. richteri* from the native range^25^. We kept loci that had at least 6 reads in all individuals (as we only have 4 individuals per social haplotype).

Using these datasets, we calculated Tajima’s D separately for each social haplotype in the native and in the introduced ranges using VCFtools^39^. The values were computed in a sliding window of 100Kbp along chromosome 16, which contains the social supergene. The total number of SNPs used in the analysis were 2,877 and 1,237 in the RAD-seq native *SB* and *Sb* haplotypes, respectively, and 24,671 and 21,004 in the fully sequenced introduced *SB* and *Sb* haplotypes, respectively. The significance of the results was assessed assuming the β-distribution^36^.

## Results

Evolutionary distances between social chromosome haplotypes were measured from a multiple genome alignment of seven *SB* and seven *Sb S. invicta* haplotypes, all from the introduced range in North America, and one *S. fugax* genome as an outgroup. These were all independent de novo assemblies of full genomes, each sequenced from a single haploid male. The distances were calculated only based on the genomic sequences aligned to the non-recombining region of the social chromosome. A second analysis used RAD sequences of 12 workers, which were homozygous at the social chromosome. These included four *SB*/*SB invicta*, four *SB*/*SB richteri* and four *Sb*/*Sb richteri*, all from the native range in South America. Again, the evolutionary distances were measured only between the sequences that were aligned to the non-recombining region of the social chromosome.

### An accelerated evolutionary rate in the Sb social variant

**Figure 1:**
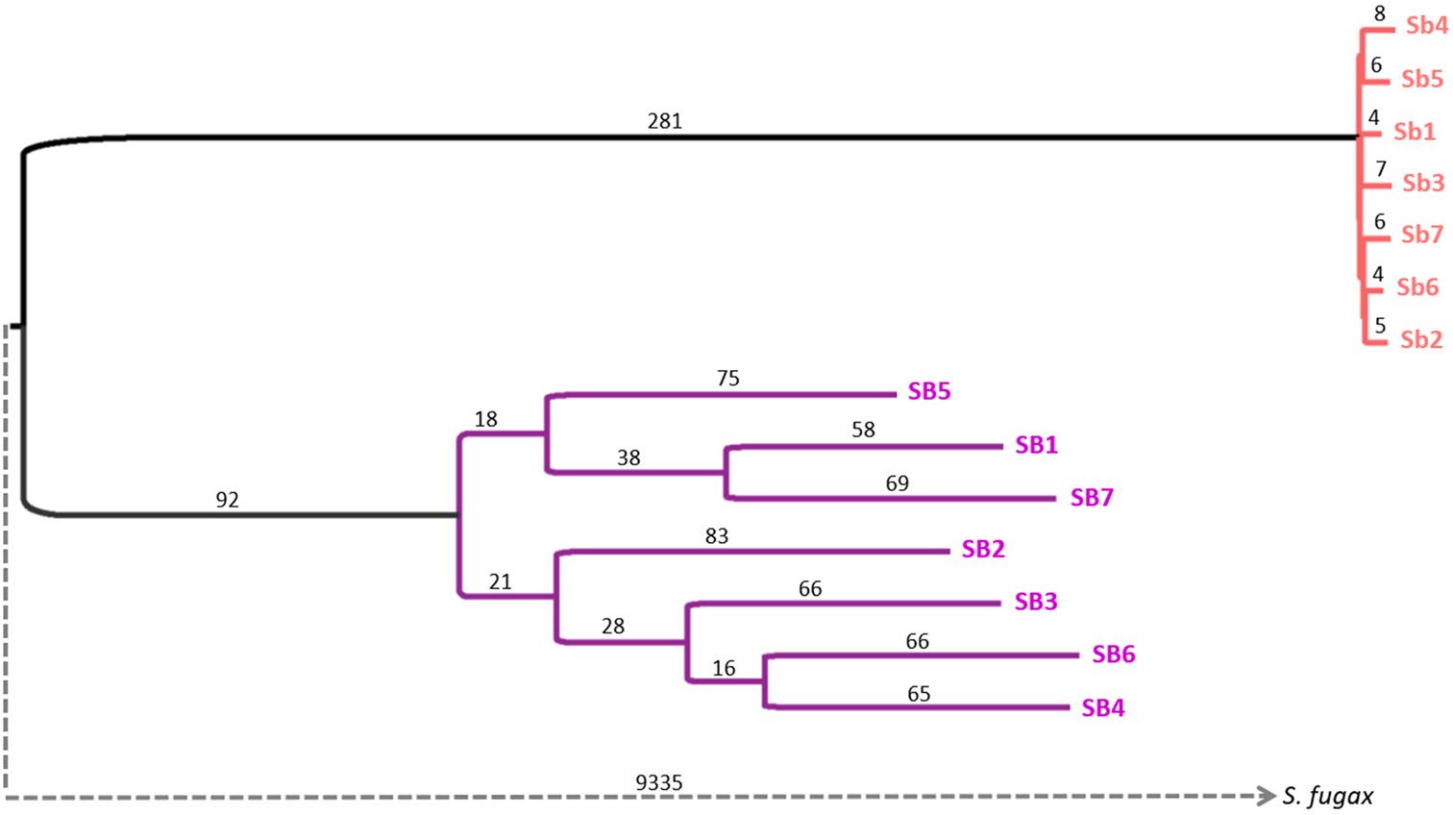
A Maximum Likelihood tree constructed from the non-recombining region of the social chromosome (in chromosome 16) in 14 introduced *S. invicta* samples. The homologous sequence in *S. fugax* genome was used as an outgroup to root the tree but the branch leading to it is only illustrated in order to maintain the proportional representation of *SB* and *Sb* branches. Branch lengths are the average number of substitutions per 100,000 sites.

The derived *Sb* haplotype evolves at an accelerated rate in comparison to the *SB* variant, as inferred from the maximum likelihood tree constructed from the fully sequenced haplotypes (Fig. 1). The lengths of the paths from the divergence point of *SB* and *Sb* to the *Sb* haplotypes are 2.9 ± 0.01 substitutions per 1,000 sites (average and standard deviation), while the path lengths to the *SB* haplotypes are 2.1 ± 0.14, which is significantly shorter (*p* = 0.002 in a two tailed Wilcoxon test). Thus, the evolutionary rate of the *Sb* sequences is accelerated by the factor of 1.4 ± 0.2 relative to the rate of the *SB* sequences.

### Dating the formation of the supergene

The phylogeny reconstructed for the social chromosome haplotypes and the *S. fugax* outgroup allowed us to calculate a ratio of 1:23 between *SB*–*Sb* divergence time and *S. invicta* and *S. fugax* speciation time. This was done by comparing the average distance measured from an *SB* haplotype to the common ancestor of *SB* and *Sb* and the average distance from an *SB* haplotype to the common ancestor of *S. invicta* and *S. fugax*. This ratio is not affected by the accelerated evolutionary rate of the *Sb* variant because it is not part of these paths in the tree (Fig. 1). Given that the two species diverged 25 (95% confidence interval 18–32) MYA^29^, the divergence between the social morphs is estimated at 1.1 (0.7–1.6) MYA.

### Possible introgression of the Sb haplotype across species

**Figure 2:**
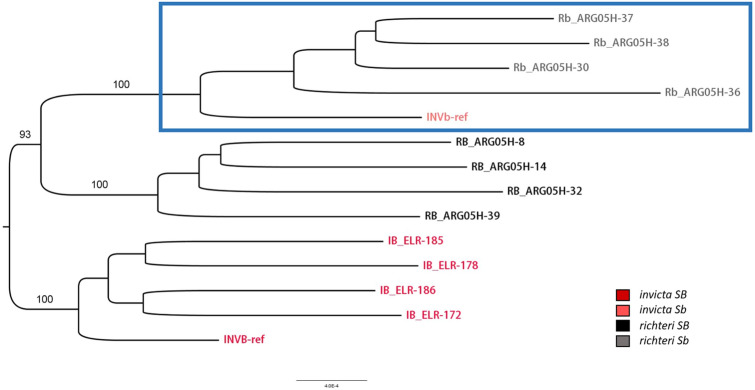
A Maximum Likelihood tree constructed from the RAD-seq of the non-recombining region in 12 native *S. invicta* and *S. richteri* samples. The *two S. invicta* reference genomes (marked *INVB-ref* and *INVb-ref*) are also included. The monophyletic subtree of the *Sb* haplotypes is marked by a blue box. The homologous sequence in *S. fugax* genome was used as an outgroup to root the tree but is not shown.

In the phylogenetic tree constructed for the RAD sequenced samples (Fig. 2), the first split is between the *invicta SB* and *richteri SB*, and the subsequent split is between the *SB* and *Sb* haplotype groups, which implies that speciation preceded the formation of the supergene. We also included in this phylogeny the *invicta SB* and *Sb* reference genomes to represent both haplotypes in both species. Thereby, we can observe that the *Sb* haplotypes of both species form a monophyletic clade within the *richteri* clade. This suggests that the derived *Sb* haplotype evolved within the *richteri* lineage and was then introgressed into the *invicta* lineage.

**Figure 3:**
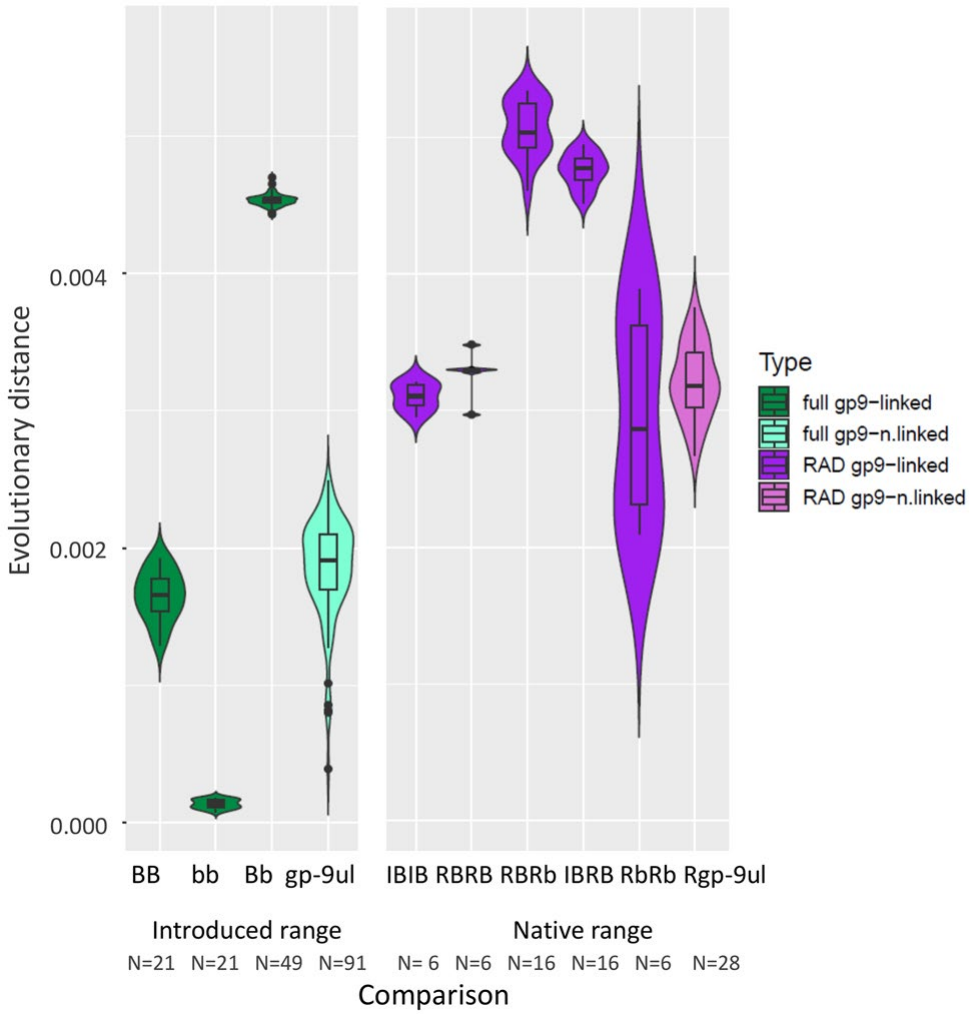
Distribution of pairwise evolutionary distances among several groups of haplotypes, summarized in violin plots. The distances were measured between the whole-genome sequences of non-recombining region in 14 *S. invicta* individuals sampled in the introduced range (green) and between RAD-sequences in the same genomic region of four *S. invicta* and eight *S. richteri* individuals sampled in the native range (purple). The evolutionary distances measured in the genomic region of chromosome 16 that is outside the non-recombining region are also shown for comparison (light green and light purple). The number of pairwise comparisons is indicated under each plot. Introduced *S. invicta* genomes: BB–*SB* vs. *SB*; bb–*Sb* vs. *Sb*; Bb–*SB* vs. *Sb*.; *gp-9ul*–all 14 individuals compared over their *gp-9* unlinked genomic region. RAD sequenced genomes: IBIB–*invicta SB* vs*. invicta SB*; RBRB–*richteri SB* vs. *richteri SB*; RBRb–*richteri SB* vs. *richteri Sb*; IBRB–*invicta SB* vs. *richteri SB*; RbRb–*richteri Sb* vs. *richteri Sb*; *Rgp-9ul*–all 8 *richteri* individuals compared over their *gp-9* unlinked genomic region.

Figure 3 presents the distributions of pairwise distances between haplotype groups in either whole genome or RAD-seq datasets. The analysis of the native *S. invicta* and *S. richteri* resulted in slightly longer distances between *richteri SB* and *richteri Sb* (5.1 ± 0.2 substitutions per 1,000 sites) than those measured between *invicta SB* and *richteri SB* haplotypes (4.8 ± 0.1). However, since the *Sb* haplotypes evolve 1.4 fold faster than *SB*, then the inferred time since the speciation is actually longer than the time since haplotype divergence (*p* < 5 × 10^–6^ in a two tailed Wilcoxon test). The distance between *richteri SB* and *richteri Sb* is composed of two components: 5.1 = *t r*_*B*_ + *t r*_*b*_, where *t* is the time since supergene formation and *r*_*B*_ and *r*_*b*_ are the evolutionary rates in *SB* and *Sb* respectively. This means that 5.1 = *t r*_*B*_ + 1.4*t r*_*B*_ and so *t* = 2.1/*r*_*B*_. The time since *invicta SB*’s and *richteri SB*’s divergence is 2.4/*r*_*B*_, which is greater than 2.1/*r*_*B*_. This result suggests that the formation of the supergene occurred only after the speciation of *S. invicta* and *S. richteri*, which is in agreement with the phylogeny in Fig. 2.

### Divergence levels within haplotype groups in the native and introduced ranges

In general, the distances within haplotype groups in the fully sequenced *S. invicta*, sampled in the introduced range, are shorter than in the equivalent groups in the RAD sequenced *S. invicta* and *S. richteri*, sampled in the native range (Fig. 3). These results are consistent with a recent population bottleneck event, as expected with a recently introduced population. Most striking are the differences in the mean and variance of the distances measured within the *Sb* haplotype group in the native range (*RbRb*; 3 ± 0.8 substitutions per 1,000 sites) compared to the distances measured within the *Sb* group in the introduced range (*bb*; 0.1 ± 0.03), which is more than one order of magnitude. The former group is the most diverse and latter—the least diverse among all haplotype groups. Conversely, the diversity of the *SB* haplotype group in the introduced range (1.7 ± 0.2) is reduced by only ~ 50% relative to the corresponding native range groups (3.1 ± 0.1; 3.3 ± 0.2 for *invicta* and *richteri*, respectively). Pairwise distances and their summary statistics can be found in Supplementary Tables S1A,B and S2A,B.

### Tajima’s D statistics over chromosome 16

**Figure 4:**
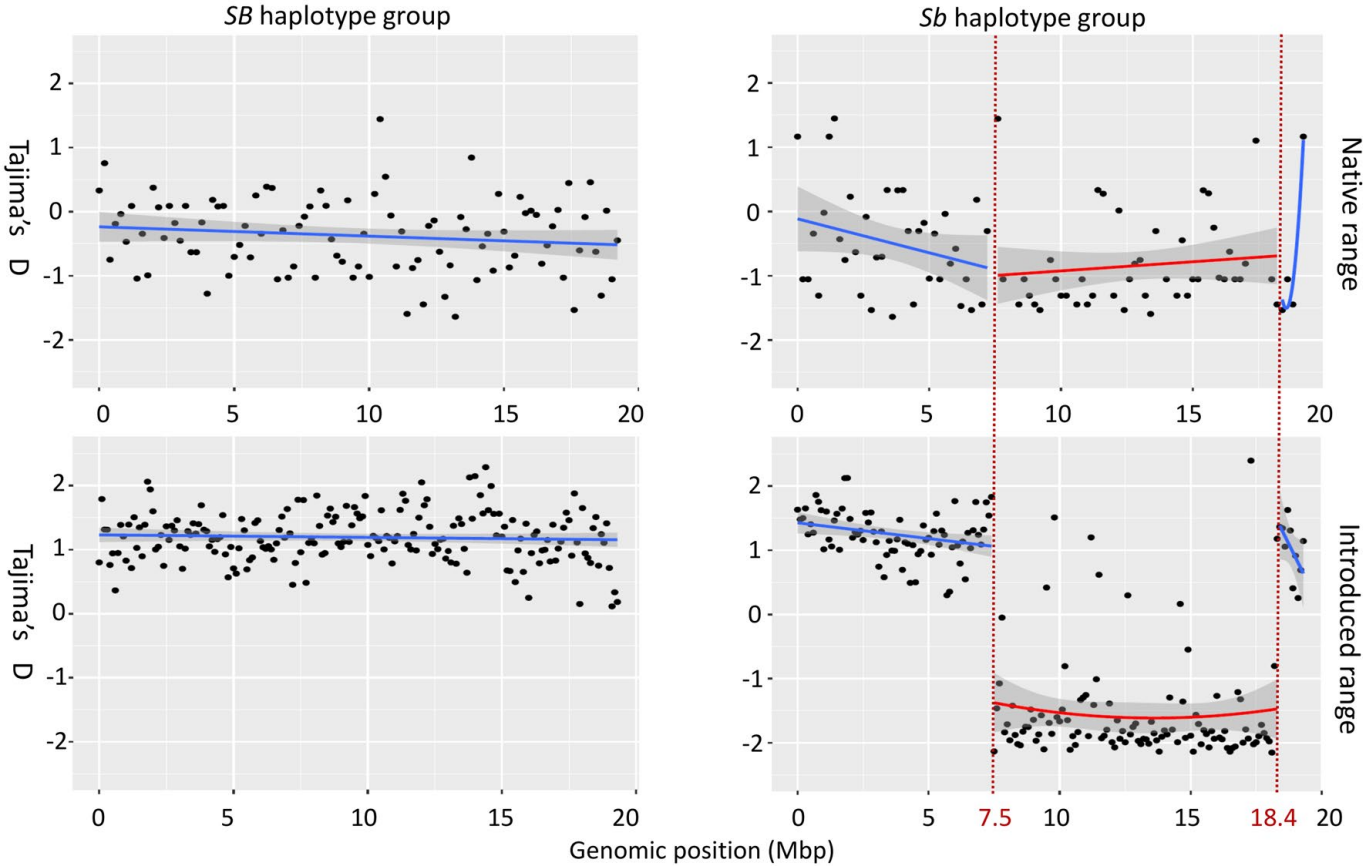
Tajima’s D statistic for 100 Kb windows across chromosome 16 for native range *S. richteri* (RAD-seq data) and introduced ranges *S. invicta* (whole-genome data) for each social haplotype group. Lines and grey zone represent smoothing with the linear model method over 95% confidence levels. A 10.9 Mbp long segment, that for the introduced range *Sb* haplotypes contains all windows with negative values, including 82 windows with significantly negative values, was smoothed separately in the *Sb* haplotype groups (positions 7.5–18.4 Mbp in red).

We calculated Tajima’s D statistics for different haplotype groups in a 100Kbp sliding window along chromosome 16, which contains the social supergene (Fig. 4). In the *Sb* haplotype group of the introduced range (whole genome data; n = 6), we find a segment of 109 consecutive windows (total length of 10.9 Mbp), with the mean Tajima’s D value of − 1.51, which is significantly different from zero. This segment, which overlaps with all of the scaffolds identified to be linked to the social chromosome, includes 101 windows with negative values, 82 of which were significantly negative. Outside of this region, and for the entire chromosome 16 in the introduced *SB* haplotype group, there were no negative windows and only a small number of significantly positive windows (2 and 8 respectively). In the same 10.9 Mbp region in the native *Sb* haplotype group (RAD-seq data; n = 4), 29 windows were significantly negative and the mean Tajima’s D was − 0.842 across it. There were 16 significantly negative windows outside of this region, and a total of 21 significantly negative windows across the entire chromosome of the *SB* haplotype group. There were no windows with significantly positive Tajima’s D values in either haplotype group in the native range.

## Discussion

Our estimate of 1.1 (0.7–1.6) MYA is a much older date for the divergence of *Solenopsis* social variants than the previous estimate of 390,000 (350,000–420,000) years ago^17^. This previous dating was made relative to the divergence time of two leaf cutter ant species in a different tribe (tribe *Attini*), whereas our estimate was made relative to the divergence from another species in the *Solenopsis* genus (tribe *Solenopsidini*). Additionally, the dating of *S. fugax*–*S. invicta* divergence time is based on more robust phylogenetic dating informed by 27 fossil calibration points^29,40^ relative to only three in the previous study. We previously estimated the number of generations since gene flow between *S. invicta* and *S. richteri* ceased at 190,000 (100,000–350,000), using a maximum likelihood coalescence analysis of 15,040 RAD-seq loci^25^. Assuming a generation time of six years, this means that the species separated 1.1 (0.6–2.1) MYA. This time frame overlaps with the above estimate for the age of the supergene and therefor does not resolve the order of these evolutionary events. We note that there is uncertainty in the generation time parameter, and since it may be as short as three years^41^, the estimate for the speciation may only be 0.55 MYA.

In our phylogenic analysis of the non-recombining region in native *S. invicta* and *S. richteri* samples, the monophyletic clade of *Sb* haplotype of both species was assigned to the same subtree as the *richteri SB* haplotypes with bootstrap support of 93%. Thus, it seems likely that *S. invicta* had obtained its *Sb* variant through admixture with *S. richteri* or a species which is closely related to *S. richteri*. This, combined with the dating results, suggests that the formation of the supergene occurred around the same time in which the process of speciation between *S. invicta* and *S. richteri* was taking place. The *Sb* haplotype may have first evolved in one incipiently speciating population and then introgressed into the other populations before the reproductive barrier between them was completely formed.

Recently, Yan et al.^20^ conducted a phylogenetic dating analysis of eight *Solenopsis* species using five conserved genes. They estimated the radiation time of the social polymorphic species at 0.5 (0.1–1.1) MYA, which is in the lower range of our abovementioned estimate for the speciation of *S. invicta* and *S. richteri*. They also estimated the divergence time of the *Solenopsis* socially polymorphic clade from *S. saevissima*, which does not harbor the *Sb* haplotype, at 0.7 (0.1–1.5) MYA. This speciation event should be taken as an upper-bound for appearance of the social chromosome, and fits within the lower range of our dating of the supergene. It should be noted that speciation between two incipient species is only complete when a reproductive barrier is formed between them, preventing the exchange of genes (with the possible exception of isolated mating and introgression). This is true for the speciation dating from our previous study. On the other hand, the evolutionary distances inferred from the divergence of genes or haplotype sequences corresponds to the time since the coalescent event for these alleles, which predates the time of reproductive isolation. Taken together, the dating estimates by Yan et al. and our own suggest that the supergene was formed approximately at the same time of the speciation of the socially polymorphic clade.

Gotzek et al.^42^ anayzed sequences of the *gp-9* gene from 14 *Solenopsis* species, including the six species of the *Solenopsis* socially polymorphic clade, and found that the monophyletic *gp-9*^*b*^ clade is most closely related to *gp-9*^*B*^ allele sequences of *S. invicta* with very high support (bootstrap of 97%), while the *gp-9*^*B*^ clade is not monophyletic across the socially polymorphic species. This implies that, contrary to our results, *gp-9*^*b*^ has originated in *S. invicta* and introgressed into the other species. On the other hand, Yan et al. in their analysis of the entire social chromosome, have presented three different social haplotype trees, depending on different filtering criteria. In one, the *SB* and *Sb* haplotypes form separate monophyletic clades (albeit with bootstrap of only 47% to the monophyly of *SB*), which suggests that the *Sb* haplotype already existed in an ancestral species to the clade. In the other two trees, the *SB* haplotype clade is paraphyletic with high bootstrap support, and so these trees support the introgression scenario (extended data). Moreover, in one of them, the likely species of origin of the *Sb* haplotype is *S. richteri*, similar to our results. Altogether, the inconsistent tree topologies from the various studies indicate that the two evolutionary processes of speciation and social haplotypes divergence, which were probably only established over the course of many generations, most likely overlapped. Fast species radiation and incomplete lineage sorting may also account for the ambiguity of the results. Such evolutionary dynamics would result in different tree topologies for different haplotype samples and for different genes or genomic regions within the supergene.

Cohanim et al.^43^ investigated natural selection on protein-coding regions of the social chromosome and calculated the ratio of non-synonymous substitutions (*dN*) to synonymous substitutions (*dS*). The ratio in the *Sb* haplotype (*dN*_*b*_/*dS*_*b*_) was estimated at 0.209, while in the *SB* haplotype (*dN*_*B*_/*dS*_*B*_) it was 0.087, implying that much stronger purifying selection pressures act on the *SB* haplotype. In our analysis, the 1.4-fold acceleration in the evolutionary rate of the *Sb* haplotype compared to the *SB* haplotype was calculated over mostly non-coding genomic regions (92% of the supergene is non-coding sequences according Cohanim et al*.*). Therefore, the higher substitution rate we report here is probably composed of elevated rate in both amino-acid substitutions and substitutions in non-coding elements (e.g. untranslated regions in exons, promoters, transcription factor binding sites). The elevated rates likely reflect differences in natural selection pressures over the social haplotypes, acting both on amino acid and on mutations in non-coding elements. These differences can be explained by the reduced efficiency of purifying selection on functional sites due to the lack of recombination in *Sb*, and possibly also by positive selection acting on adaptive traits during the evolution of the polygyne form.

The within-group diversity levels in the native range do not significantly differ between *richteri Sb* and *SB* haplotype groups (*p* > 0.9 in a two tailed Wilcoxon test), or between the *gp-9* unlinked genomic region and either of the haplotype groups (*p* > 0.4 in both cases). However, the evolutionary distances measured among the *Sb* haplotypes vary greatly, with almost an order of magnitude larger standard deviation in the *Sb* haplotypes group compared to the *SB* haplotypes group. As *Sb* is almost only found in *SB*/*Sb* polygyne queens (*Sb*/*Sb* queens rarely reach reproductive maturity), recombination between the *Sb* haplotypes is rare, resulting in increasingly separating *Sb* lineages. The varying evolutionary distances directly reflect different coalescent times between pairs of *Sb* haplotypes. Conversely, *SB* haplotypes do recombine in *SB*/*SB* queens, averaging the distances between different haplotypes as shown by the more uniform diversity levels among the *SB* haplotypes.

In contrast to the diversity of native range *Sb* haplotypes, in the introduced range, *Sb* diversity level is much reduced. The distances among the *Sb* haplotypes in the introduced range are all much lower than the lowest pairwise distance between *Sb* haplotypes in the native range, which is consistent with a single clone of *Sb* haplotypes that passed through the bottleneck. This lack of diversity was already pointed out by Pracana et al.^44^ when analyzing the same introduced *S. invicta* individuals used in our study. Pracana et al. suggested two possible explanations to the low diversity levels. The first was a recent selective sweep of a single *Sb* haplotype. As we found that the diversity levels of the native *richteri Sb* do not differ from those of the native *richteri SB*, this seems unlikely. The second was that polygyny was only introduced to the USA as late as the 1970s in a secondary introduction event, resulting in severe bottleneck effect of the introduced *Sb* haplotype. This can account to its much reduced diversity compared to the diversity of introduced *SB* haplotype, which is the accumulated result of all introductions. Furthermore, the lower fitness of *Sb* males^16,45^ means that a mated polygyne queen most likely carried two *SB* haplotypes and only one *Sb* haplotype into the introduced range. Even if the founding colony consisted of many polygyne queens, they may have contributed only one clone of *Sb* haplotypes if they were all maternally related. Negative Tajima’s D values in the supergene, which are the result of increased number of rare alleles, may also be interpreted as the effect of a population bottleneck. We conclude that effectively only a single *Sb* haplotype passed the bottleneck of the introduction and there is no need to invoke selection to explain the very low diversity and the excess of rare alleles in this haplotype following the recent introduction.

## Conclusion

We dated the supergene of the *Solenopsis* social chromosome at 1.1 (0.7–1.6) million years, which is substantially older than previous estimates. The appearance of the derived polygyne social structure has likely occurred concomitantly with the speciation of *S. invicta* and *S. richteri*, during the gradual formation of barriers to gene flow between them. We have compared the evolutionary rate of the two social variants and found that the *Sb* haplotype evolves at a rate which is 1.4 times that of the *SB* haplotype, likely due to the suppression of recombination in the *Sb* haplotypes. While the levels of diversity within the native *SB* haplotypes and within the native *Sb* haplotypes are similar, the highly variable evolutionary distances among the latter mark the separately evolving *Sb* clones. This is in stark contrast to the much lower diversity levels of the *Sb* haplotype in the introduced range, which is likely the result of a very severe bottleneck that effectively allowed only a single close of *Sb* haplotypes into the introduced population.

## Supplementary information

Supplementary file1 (DOCX 28 kb)
