# Supplementary material for: The social supergene dates back to the speciation time of two Solenopsis fire ant species
Source: Sci Rep. 2020 Jul 14;10:11538. doi: 10.1038/s41598-020-67999-z (PMC7360596; doi:10.1038/s41598-020-67999-z)
Supplement: Supplementary file 1 — Supplementary file1 (DOCX 28 kb) [file 41598_2020_67999_MOESM1_ESM.docx]

# **The social supergene dates back to the speciation time of two *Solenopsis* fire ant species**

Cohen Pnina, Cohanim Amir Bar, Privman Eyal#

Department of Evolutionary and Environmental Biology, Institute of Evolution, University of Haifa, Haifa, Israel.

Table S1. Evolutionary distances between 14 introduced *S. invicta*

A

B

| Comparisons  between | Number of comparisons | Mean | SD | Min | Max |
| --- | --- | --- | --- | --- | --- |
| *SB* *invicta*  *SB*  *invicta* | 21 | 0.00165 | 0.00018 | 0.00129 | 0.00192 |
| *Sb invicta Sb invicta* | 21 | 0.00013 | 0.00003 | 0.00008 | 0.00018 |
| *Sb invicta*  *SB*  *invicta* | 49 | 0.00453 | 0.00005 | 0.00443 | 0.0047 |
| gp9-unlinked *invicta* | 91 | 0.00185 | 0.00037 | 0.00039 | 0.00249 |
| *S. fugax*  *SB*  *invicta* | 7 | 0.0752 | 0.00066 | 0.07388 | 0.07581 |
| *S. fugax*  *Sb*  *invicta* | 7 | 0.07611 | 0.00032 | 0.07557 | 0.07644 |

Complete (A) and summary (B) statistics of evolutionary distances measured in pair-wise comparisons between 14 fully sequenced *S. invicta* of the *SB* and *Sb* haplotypes sampled in the introduced range, and a fully sequenced *S. fugax*.

Table S2. Evolutionary distances between 12 native *S. invicta* and *S. richteri*

A

B

| Comparisons between | Number of comparisons | Mean | SD | Min | Max |
| --- | --- | --- | --- | --- | --- |
| *SB* *invicta*  *SB invicta* | 6 | 0.00310 | 0.00010 | 0.00296 | 0.00321 |
| *SB* *richteri*  *SB richteri* | 6 | 0.00327 | 0.00017 | 0.00297 | 0.00348 |
| *Sb richteri*  *Sb* *richteri* | 6 | 0.00295 | 0.00079 | 0.0021 | 0.00389 |
| *SB* *richteri*  *Sb* *richteri* | 16 | 0.00507 | 0.00021 | 0.00461 | 0.00534 |
| *SB invicta*  *SB* *richteri* | 16 | 0.00476 | 0.00013 | 0.00452 | 0.00494 |
| gp9-unlinked *richteri* | 28 | 0.00321 | 0.00027 | 0.00267 | 0.00375 |

Complete (A) and summary (B) statistics of evolutionary distances measured in pair-wise comparisons between four RAD sequenced *SB invicta*, four *SB* and four *Sb* *richteri* sampled in the native range.
